# Hepatitis E Virus (HEV) in Heavy Pigs in Slaughterhouses of Northern Italy: Investigation of Seroprevalence, Viraemia, and Faecal Shedding

**DOI:** 10.3390/ani13182942

**Published:** 2023-09-16

**Authors:** Marina Monini, Ilaria Di Bartolo, Luca De Sabato, Giovanni Ianiro, Francesca Agostinelli, Fabio Ostanello

**Affiliations:** 1 Department of Food Safety, Nutrition and Veterinary Public Health, Istituto Superiore di Sanità, Viale Regina Elena, 299, 00161 Rome, Italy; marina.monini@iss.it (M.M.); luca.desabato@iss.it (L.D.S.); giovanni.ianiro@iss.it (G.I.); francesc.agostinell5@studio.unibo.it (F.A.); 2 Department of Veterinary Medical Sciences, University of Bologna, Via Tolara di Sopra, 50, Ozzano dell’Emilia, 40064 Bologna, Italy; fabio.ostanello@unibo.it

**Keywords:** HEV, zoonoses, pigs, slaughterhouse, viraemia, IgM, seroprevalence, ELISA

## Abstract

**Simple Summary:**

Hepatitis E virus (HEV) is spread worldwide among both humans and animals. In humans, the diseases can be asymptomatic, but it can also lead to chronic hepatitis, especially in immunocompromised patients. In the European Union, most human cases are foodborne and caused by the zoonotic genotypes HEV-3 and HEV-4. Pigs and wild boars serve as the main reservoirs for these zoonotic genotypes, as they are frequently infected by the virus, which replicates in the liver and is released in their faeces. This study aimed to assess the presence of HEV in heavy pigs (>160 kg) in a large abattoir in Italy. Both 240 pooled faecal samples collected on 24 trucks after unloading pigs and 88 plasma samples collected individually were negative for HEV RNA. Only five pigs (1.9%) tested positive for IgM, a sign of recent infection. Conversely, a high seroprevalence of 89.2%, confirmed by detection of total anti-HEV antibodies, demonstrated the wide exposure of pigs to the virus.

**Abstract:**

Hepatitis E virus (HEV) is considered an emerging threat in Europe, owing to the increased number of human cases and the widespread presence of the virus in pigs at farms. Most cases in industrialized countries are caused by the zoonotic HEV-3 genotype. The main transmission route of HEV-3 in Europe is foodborne, through consumption of raw or undercooked liver pork and wild boar meat. Pigs become susceptible to HEV infection after the loss of maternal immunity, and the majority of adult pigs test positive for IgG anti-HEV antibodies. Nonetheless, HEV-infected pigs in terms of liver, faeces, and rarely blood are identified at slaughterhouses. The present study aimed to investigate the prevalence of HEV-positive batches of Italian heavy pigs at slaughterhouses, assessing the presence of animals still shedding HEV upon their arrival at the slaughterhouse by sampling faeces collected from the floor of the trucks used for their transport. The occurrence of viraemic animals and the seroprevalence of anti-HEV antibodies were also assessed. The results obtained indicated the presence of anti-HEV IgM (1.9%), and a high seroprevalence of anti-HEV total antibodies (IgG, IgM, IgA; 89.2%, *n* = 260). HEV RNA was not detected in either plasma or faecal samples. Nevertheless, seropositive animals were identified in all eight batches investigated, confirming the widespread exposure of pigs to HEV at both individual and farm levels. Future studies are needed to assess the factors associated with the risk of HEV presence on farms, with the aim to prevent virus introduction and spread within farms, thereby eliminating the risk at slaughterhouse.

## 1. Introduction

Hepatitis E is an acute viral disease caused by the RNA hepatitis E virus (HEV). The virus has recently been classified into two subfamilies: *Parahepevirinae*, which exclusively infects only trout and salmon, and *Orthohepevirinae*, which infects birds and mammals. The species *Paslahepevirus balayani* includes viruses classified into eight genotypes, based on their nucleotide heterogeneity and host specificity [1]. Among these, HEV-1 and HEV-2 genotypes infect humans, HEV-3, HEV-4, and HEV-7 are zoonotic, infecting humans and animals. HEV-5, HEV-6, and HEV-8 have only been detected in animals. HEV-3 and HEV-4 cause self-limited acute hepatitis of zoonotic origin in humans and infect several mammalian species, including swine, wild boar, and, to a lesser extent, deer, rabbits, and roe-deer [2]. In Europe, HEV-3 is the most frequently detected genotype in both humans and animals [3,4]. HEV-3 transmission occurs through foodborne routes, primarily via the consumption of contaminated foods of animal origin such as pork liver sausages and wild boar meat, often eaten raw or undercooked [3,4]. Professional risk of infection exists for exposed workers, as evidenced by higher anti-HEV seroprevalence in farmers, veterinarians, slaughterers, hunters, and forestry workers, when compared to the general population [5,6,7].

Pigs serve as the main reservoirs of HEV-3 and HEV-4 [4,8]. The infection in pigs is typically asymptomatic, and the presence of anti-HEV antibodies is most commonly observed in pigs older than 3 months. Studies have indicated that the virus is widespread on pig farms worldwide (America, Europe, and Asia) [9,10].

Pigs become infected through direct contact with faeces or other body secretions of HEV-positive pigs [11], typically early in their life after the loss of maternal immunity (2–3 months of age) [5,8]. Following infection, pigs experience a transient viraemia lasting approximately 1–2 weeks, while the viral shedding in faeces can persist for up to 6 weeks [11]. The virus primarily replicates in the liver and is subsequently released in the bile. The seroconversion begins with a decrease in faecal shedding around 3–4 weeks after exposure, marked by the appearance of IgM, followed by IgG. The duration of seroconversion is unknown, but it is believed that pigs can be reinfected throughout their lives [4,10] as HEV-positive adult pigs (older than 5 months) have been observed. This dynamic of infection explains why weaner pigs are the age category most susceptible to the infection, with susceptibility decreasing as they age [12,13,14]. The seroprevalence increases in adult animals [12,15], corresponding to an increase in weight (which corresponds to the age group) [16]. However, a small number of pigs at slaughter age still test positive for HEV presence in liver (0.25–11.0% in adults) [14,17,18,19,20,21,22], faeces [18,21,22,23], and very rarely in muscle [12,18]. The presence of the virus decreases with the age of animals, with higher rates in younger pigs and a significantly high percentage in the liver at the abattoir only in pigs aged 1–3 months [24]. The earlier age of slaughtering is considered a risk factor that increases the number of animals still being positive for HEV presence in the liver [25]. Information on viraemia is limited, with only a few studies reporting the presence of HEV RNA in pig blood at slaughterhouses, primarily in young pigs [12,17,20,26,27]. Viraemia and the presence of IgM are frequently associated [20], and the presence of IgG does not exclude the presence of IgM, indicating the existence of a complex dynamic of infection that can vary considerably.

In Italy, the virus is widespread in pig farms, particularly from Northern Italy where the majority of pigs are housed in intensive farms with herd prevalence ranging from 24.8% to 52.9% [7,28,29]. However, it is also prevalent in small farms, which are more common in the south of the country, with farm prevalence ranging from 12.5% to 50% [30,31]. At slaughterhouses, the virus has been detected in various tissues, including the liver, faeces, bile, and rarely in blood [12,26,32]. Moreover, in Italy, HEV has been identified in other wild, synanthropic, or domestic species, including wild boars [33,34], wolves [35], red deer [36], rats [37], goats [38], and sheep [39]. In some other hosts such as red foxes [40], chamois [41], dogs [42], cats [43], and rabbits [44] antibodies against HEV have been detected, indicating exposure to the virus.

In this study, we investigated the presence of the virus in pigs at the slaughterhouse. We collected pig faeces shortly after unloading from the trucks at the abattoir and obtained individual pig plasma samples during bleeding. Plasma samples were also screened for anti-HEV antibodies. The primary objectives of the study were to assess the risk of viraemic pigs at the slaughterhouse and to determine the extent of exposure to HEV among adult pigs during their lifespan, as indicated by the presence of antibodies against the virus.

## 2. Materials and Methods

### 2.1. Sample Collection

Samples were collected in April 2022 from a single abattoir in Northern Italy that slaughters approximately 22,000 pigs per week. The samples were obtained from 24 farms (Table 1). Over the course of two consecutive days of slaughtering, 24 trucks, which were used to transport pigs from various farms, all located in Northern Italy, were randomly selected for sampling. These pigs, which were intended for the production of raw hams such as Parma and San Daniele Prosciutto (Protected Designation of Origin), were all 9 months of age and had a bodyweight exceeding 160 kg. The sampling process involved collecting samples from each of the 24 trucks. Each truck transported animals from a single farm, constituting one batch per farm. The pigs were held for 1–3 h during their transportation from the farms to the abattoir. Table 1 provides a summary of the origin of the animals included in the study, including information about the types of farms and other relevant characteristics. Specifically, pig faeces were sampled directly from the inner part of the tractor and trailer for 20 batches, each of which involved the transportation of 111 to 135 animals per batch. Additionally, two batches were sampled in tractors, with 62 and 65 pigs transported in each batch, respectively.

Ten samples were collected from each of the 24 tractors and/or trailers immediately after the unloading of animals. Each sample was composed by pooling together 10 portions of dropped faeces collected from different areas of the floor inside the tractor or trailer.

The analysis of 10-pooled faecal samples for each batch was designed to achieve a sensitivity that would allow the detection of at least one positive sample when the within-batch prevalence was ≥4%. This assumption was based on a test sensitivity of 90% for all pooled sample sizes and aimed to provide a desired cluster sensitivity of 95% [45].

Overall, 240 pooled faecal samples were collected in sterile plastic bags and kept under refrigeration conditions (4–8 °C) and frozen at −20 °C within 2–4 h from collection. Prior to RNA extraction, pooled faecal samples (approximately 1 g) were diluted in sterile water to a final 10% (*w*:*v*) faecal suspension and clarified using centrifugation at 10,000× *g* for 10 min.

Eight batches, out of the twenty-four batches from which faecal samples were collected, were randomly selected for individual blood sampling during bleeding. These eight batches were obtained from fattener farms located in Lombardia (*n* = 4), Emilia-Romagna (*n* = 3), and Veneto (*n* = 1) Regions (Table 2). In total, 260 blood samples were individually collected from pigs randomly selected within each of the eight batches. Due to the varying number of pigs in each batch, the speed of the slaughter line varied, making it occasionally impossible to collect the same number of blood samples. Overall, in four batches, 45 blood samples were collected per batch, while in the other remaining four batches, 20 samples were collected per batch (Table 2). For each pig, blood was collected in tubes pre-filled with EDTA (Venoject, Terumo Italia, Rome, Italy) and immediately stored at 4 °C for 12 h. Plasma was recovered after centrifugation at 3000× *g* for 5 min.

### 2.2. Enzyme-Linked Immunosorbent Assay (ELISA)

Plasma samples were tested for the detection of total antibodies (IgA, IgM, and IgG) against HEV using the multi-species HEV ELISA 4.0 kits for serum or plasma samples (MP Biomedicals Asia Pacific Pte Ltd., Singapore, formerly Genelabs Diagnostics Pte), following manufacturers’ instructions. For IgM anti-HEV detection, the HEV ELISA 3.0 kit (MP Biomedicals Asia Pacific Pte Ltd., Singapore, formerly Genelabs Diagnostics Pte) for human species was used by changing the secondary antibodies and using the horseradish peroxidase (HRP)-conjugate goat anti-porcine IgM antibody (Sigma-Aldrich, St. Louis, MO, USA). Both tests utilized the recombinant highly conserved conformational epitope derived from the HEV capsid protein (ORF2).

Since positive plasma samples for IgM detection were not available to calculate the cut-off value, we conducted some preliminary assays.

To evaluate specificity of the modified ELISA 3.0 kit, 30 negative controls (plasma sample found negative on ELISA evaluation for the detection of total anti–HEV antibodies) were assayed using the work dilution (1:21 in diluent reagent) as suggested by the manufacturer’s kit. The negative controls showed an unacceptable amount of background signals (OD, optical density, values). At a 1:150 dilution, background noise associated with negative samples was diminished with no significant loss of signal.

Afterward, ELISA for the IgM detection was conducted and cut-off values were calculated by following two approaches to establish the most straightforward approach to compute cut-off values. The first followed a previous approach used by Crossan et al. [27] that adapted an ELISA designed for humans by changing the secondary antibodies and calculating the cut-off value as a mean of the OD values of negative plasma ± 3SD (SD: standard deviation). Similarly, in our test, employing a cut-off value of three SD above the mean of the negative controls, all 30 negative samples were also confirmed in the IgM ELISA. Afterward, for each plate, the mean OD values of negative plasma ± 3SD were used to calculate the cut-off point and were compared to the second approach of the change-point analysis. This second approach is based on estimating a step point of the OD values which exhibits a clear increment compared to the foregoing values, discriminating among positive (sample with an OD value major of the OD of the change-point value) and negative plasma (sample with an OD minor of the change-point value) [46]. The cut-off values were determined by the two methods for each processed microtiter plate. To increase the specificity, we decided to use the approach showing the higher cut-off values. For the change-point cut-off calculation, the changepoint.np package was used as method for nonparametric change-point detection using R software version 4.1.2: (https://www.r-project.org, accessed on 29 May 2023). 

### 2.3. HEV RNA Detection in Plasma and Faecal Samples

A total of 88 plasma samples, 11 from each of the 8 batches (*n* = 8), and 240 pooled faecal samples from 24 batches were tested for the presence of HEV RNA. Nucleic acid extractions were carried out using the Qiamp Viral Mini Kit for pooled faecal samples and plasma. This involved processing 150 μL of the supernatant from the 10% (*w*/*v*) faecal suspension and 100 μL of plasma, with elution volumes of 100 μL and 50 μL, respectively.

For HEV detection, 5 µL of the RNA sample was analysed using a broad-range HEV Real-time Reverse Transcription-PCR (RT-PCR) using the RNA UltraSense One-Step qRT-PCR System (Thermofisher Scientific, Waltham, MA, USA) as previously described [33,47]. 

### 2.4. Statistical Analysis

Among the eight farms investigated for the presence of anti-HEV antibodies, the differences in seroprevalence (total anti-HEV) were statistically analysed. The comparison was conducted across farms based on farm size, categorized as small or large depending on the median value of pigs present (2500), and by geographical location (Lombardia, Emilia-Romagna, and Veneto Regions). This analysis was performed using the Pearson Chi-Square test. Confidence intervals (95%CI) were calculated using the binomial (Clopper–Pearson) “exact” method based on the β distribution. Statistical significance was set at *p* ≤ 0.05. All statistical analyses were carried out using SPSS 28.0.0 software (IBM SPSS Statistics, Armonk, NY, USA).

## 3. Results

Two-hundred-forty pooled faecal samples from 24 batches of pigs were subjected to Real-Time RT-PCR to detect HEV RNA, and all were negative. The presence of total anti-HEV antibodies in the plasma samples was evaluated from 260 samples obtained from 8 different batches of pigs randomly selected from among the 24 batches from which pooled faecal samples were analysed (Table 2). Out of the 260 samples tested, 232 (89.2%; 95%CI: 84.8–92.7) were positive for total anti-HEV antibodies (Table 2). Seropositive animals were identified on all eight farms, with prevalence ranging from 66.7% (farm 6) to 100% (farm 4). Additionally, five samples (1.9%) from two farms also tested positive for anti-HEV IgM antibodies. Five samples (1.9%) from two farms were also positive for anti-HEV IgM. Seroprevalence difference (referring to total antibodies detection) between farms was statistically significant (χ^2^ = 32.6; *p* < 0.01) while grouping pigs according to the geographical location of the farm (Lombardia, Emilia-Romagna, and Veneto Regions) was not (χ^2^ = 2.7; *p* = 0.25). A statistically significant difference was observed by grouping farms according to their size; pigs raised in small farms (<2500 animals) showed a significantly higher seroprevalence (χ^2^ =12.6; *p* < 0.001).

Due to the low number of IgM positive plasma, no further statistical analyses were conducted.

HEV RNA was neither detected in the 240 pooled faecal samples, estimating a prevalence lower than 1.05% (95%CI: 0.03–5.72), nor in the 88 plasma tested (Table 2).

## 4. Discussion

The presence of HEV-positive pigs in slaughterhouses has previously been documented, confirming that some pigs may be still infected even if slaughtered at an age over 6 months [12,19]. Previous studies conducted in Italy and other countries have reported that livers and faeces are the matrices that most frequently test positive, with prevalence rates ranging between 5.0% and 13.0% [15,18,20]. The age of animals has frequently been identified as a risk factor that influences the prevalence of HEV in swine with higher rates observed in younger animals (<5 months) [12]. 

In the present study, samples were randomly selected. Over two consecutive days of sampling about 30 trucks were used to transport pigs, but some of these delivered animals were from the same farm. Our selection process focused exclusively on batches of pigs from distinct farms. The pigs selected for the production of Prosciutto di Parma and San Daniele belong to Italian breeds obtained from carefully selected heavy breeds. These animals are born, raised, and slaughtered within a delimited production area in Northern Italy. The designations of Parma and San Daniele denomination for Prosciutto are exclusively reserved for ham derived from animals that are 9 months old and possessing specific distinctive features recognized as “Designation of Origin” products. For the aforementioned reasons, we believe that our sampling approach not only represents a regional production but also effectively characterizes the primary pig farming industry in Northern Italy.

In our study, the presence of HEV-positive shedding pigs was investigated on 24 batches of animals, each from a distinct farm, collecting pooled faecal samples on trucks immediately after the unloading of animals at the abattoir. As proved in previous studies, the use of pooled samples for HEV testing has a sensitivity ranging between 2.0 × 10^2^ and 1.6 × 10^4^ Genome Copies/g, achieved by pooling together 1 positive and 19 negative animals [13]. Furthermore, collecting faeces during the slaughtering process may not be feasible, as it could interfere with the line speed and pose a risk of faecal cross-contamination. Therefore, the use of pools of faeces represents a useful alternative method for examining numerous samples without disrupting the slaughter procedure.

None of the 240 examined pooled faecal samples were positive for HEV. This result confirms that the likelihood of detecting HEV shedders in the faeces of heavy pigs (approximately 160 kg of body weight and 9 months of age) is low (as indicated by the present study, <1.05%). This outcome is not surprising, as previously described, the percentage of pigs still shedding the virus in their faeces at the slaughterhouse is minimal among heavy pigs [10,12,32]. Animals typically become infected on farms at the age of 2–3 months [48]. Consequently, pigs at the abattoir (mostly >6 months of age in Italy) should no longer be infected by HEV. The presence of HEV in faeces of pigs at slaughterhouse is variable. Studies conducted in Italy, where pigs are mostly slaughtered at 9 months of age, reported a prevalence of 1.9% [12], 3.7% [21], 7.3% [23], and up to 33.3% [32]. 

The only risk factor associated with a higher prevalence of HEV RNA-positive animals at slaughterhouses was reported in the study by Chelli et al. [12], where a significantly higher prevalence (16.5%) was observed in animals slaughtered in Southern Italy. This difference could be reflective of the size of farms, which tend to be smaller in the South, or the age at which slaughtering occurs, which is generally younger in the Southern regions. Similar variations are observed outside of Italy as well. For instance, in the UK, 15.0% of caecal contents tested positive in slaughtered pigs at the age of <12 months [20], while 25.6% of caecal contents were positive in pigs (5–6 months) in the Netherlands [17]. The differences observed may primarily be associated with age, as higher prevalence is often observed in faeces from younger animals [26]. However, significant variations could also be attributed to factors at farm level such as biosecurity measures, management system, and feed. Conversely, data from faeces, liver, and plasma for the detection of HEV RNA exhibit variability and are not easily comparable. The liver, being the primary organ for viral replication, is found most frequently to be positive [12,19], and it deserves particular attention at slaughterhouses. The liver can be directly used to produce sausages, consumed raw, but can also contribute to the cross-contamination of diaphragm, which is used as by-products [4]. The same applies for blood. 

However, several studies have reported viraemia in pigs at slaughterhouses, with percentages ranging between 5.7% and 14.3% in England, the USA, and Italy [12,20,26,49], and 36.2% in the Netherlands and 44.4% in Scotland [17,27]. Except for the study conducted in the USA on animals older than 6 months, the studies investigated younger pigs. This difference may explain the absence of viraemic pigs in our study, where all pigs were 9 months old (88 pigs tested across eight batches). The absence of recent infection was further confirmed by the limited presence of IgM (5 out of 260 pigs, 1.9%). This percentage is lower than those observed in previous studies (ranging from 29.0% to 47.3%) conducted in younger animals [20,26,27].

Nevertheless, 89.2% of the analysed plasma samples (232/260) tested positive for total antibodies (IgG/IgM/IgA) across all eight farms, with prevalence ranging between 66.7% and 100% at intra-farm level, confirming that animals had a frequent exposure to the virus. 

Differences in seropositivity observed among farms revealed a higher percentage on small-sized (<2500 pigs) farms compared to large-sized farms. This factor has already been described as a risk factor related to HEV seroprevalence [10,50,51,52] as the infection spreads through the faecal–oral route among pigs. Farming systems consisting of smaller-sized farms have a higher risk for HEV transmission [12,13,53], likely due to the lower biosecurity measures applied. No other factors could be statistically linked to the moderate differences in seroprevalence observed among farms.

The used ELISA kits revealed the presence of total antibodies. Among animals seropositive in the ELISA evaluation for the detection of total antibodies (IgG, IgM, IgA), excluding the five positive in IgM, we can assume that other pigs were probably anti-HEV IgG positive. This is supported by the fact that the duration of IgA and IgM is of 4–7 weeks and significantly shorter than that of IgG [54].

The infection triggers the replication of the virus in the liver and its circulation in blood (viraemia), either simultaneously or shortly after the appearance of IgM and IgA antibodies, followed by the IgG synthesis, which are known to provide protective effects throughout the animal’s life [10,48]. However, it is important to note that the presence of antibodies, whether IgM/IgA or IgG, which indicate recent or past infections, can occur in both viraemic and non-viraemic pigs [26]. The presence of IgG and/or IgM is not definitely an indication of viraemic animals since the presence of HEV is not clearly associated with the presence of antibodies [20]. The presence of IgM in non-viraemic pigs suggests that the early IgM response may persist longer than viraemia. Subsequently, the five IgM positive pigs, assessed in this study as non-viraemic, could potentially have still been infected with HEV (positive in liver or faeces). Unfortunately, in the absence of an evaluation of the liver or faeces, we cannot confirm this hypothesis.

Viraemia is usually observed during a relatively short period after infection, whereas the IgG antibody response is generally detected around two weeks after infection and persists until slaughter [54,55]. 

The presence of viraemic pigs is a concern for pork safety because blood may contain infectious viruses, posing a risk of food contamination and a potential risk of foodborne exposure for humans [4]. A few studies were conducted to evaluate the presence of viraemic pigs at slaughterhouses [12,17,20,26,27,49]. Modelling based on available data suggests that viraemic pigs, entering an slaughterhouse, represent the main risk for consumers [56]. The significance of viraemia in animals at abattoirs is associated with a risk of meat contamination from improper bleeding. However, the level of HEV RNA contamination in meat is generally low, even in pigs with a high viral load in liver, the main organ for virus replication [19]. It is worth noting that few reports on HEV-positive muscles observed in animals younger than 5 months of age are present [12]. The absence of viraemia and HEV RNA detection in this study is promising, suggesting that age of animals probably prevents presence of infected pigs at an abattoir.

So far, the presence of anti-HEV IgM in swine sera or plasma has been rarely investigated, and no commercial ELISA, specifically designed for pigs, is available. Most studies have used commercial ELISA for the detection of IgM in humans, adapted to detect antibodies in pigs [27,57]. However, in the absence of known positive and negative plasma/sera to use as controls, preliminary assays are needed to establish the cut-off value of the modified ELISA.

We observed that when calculating the cut-off value using the change-point method, which is an alternative approach in the absence of controls [46], and the OD + 3SD, 13 (5.0%) and 10 (3.8%) IgM-positive samples were detected, respectively. Only five plasma samples were positive using both methods and were confirmed by selecting, from each plate, the higher cut-off value between the two methods. With this approach, we prioritized higher specificity over sensitivity.

## 5. Conclusions

Preventing the slaughter of infected pigs presents challenges as there are no associated symptoms that can promptly identify infected pigs, and there is no single definitive test for identifying positive animals. While IgM and IgA could serve as indicators of viraemia, their absence does not guarantee that an animal is not infected, as viruses are often present in seronegative pigs [27]. 

Risk reduction strategies should include measures to prevent cross-contamination of carcasses during evisceration by faeces or bile, thereby reducing the risk of contamination of pork products [4]. 

Furthermore, risk mitigation can be achieved by preventing infected pigs from entering the slaughter line, thereby reducing the prevalence of infection in farms.

Further studies should be conducted at slaughterhouses to assess the percentage of infected pigs, determine the average viral load in infected organs or matrices, and estimate the extent of virus dissemination during slaughter procedures. These results will enable a quantitative assessment of the HEV risk.

## Figures and Tables

**Table 1:** Description of farms of origin of animals from which faeces were tested and their geographical position, size, and type.

Region	No. of Housed Pigs	Type of Farms	
		Farrow-to-Finish	Finisher
Emilia-Romagna	2000		1
	2001–3000		2
	3001–5500		2
	>5500		2
Total		0	7
Lombardia	≤2000		2
	2001–3000	1	3
	3001–5500		2
	>5500	1	2
Total		2	9
Piemonte	>5500	0	1
Total		0	1
Veneto	≤2000		3
	3001–5500	1	1
Total		1	4
Total farms		3	21

**Table 2:** Summary of seroprevalence (*n* = 260) and results of qRT-PCR for HEV detection (*n* = 88).

Farm id.	Region	Size of Farm ^a^	No. Plasma Tested for Farm	HEV Total Antibodies (% ^b^; 95%CI)	HEV IgM (% ^b^; 95%CI)	qRT-PCR Results ^c^
2	Lombardia	small	45	43 (95.6; 84.9–99.5)	0	0
3	Lombardia	small	45	40 (88.9; 75.9–96.3)	0	0
4	Emilia-Romagna	small	45	45 (100; 92.1–100)	0	0
6	Emilia-Romagna	large	45	30 (66.7; 51.1–80.0)	2 (4.4; 0.5–15.1)	0
12	Lombardia	large	20	18 (90.0; 68.3–98.8)	2 (10.0; 1.2–31.7)	0
13	Emilia-Romagna	large	20	19 (95.0; 75.1–99.9)	0	0
14	Emilia-Romagna	large	20	18 (90.0; 68.3–98.8)	0	0
22	Veneto	small	20	19 (95.0; 75.1–99.9)	1 (5.0; 0.1–24.9)	0
Total			260	232 (89.2; 84.8–92.7)	5 (1.9; 0.6–4.4)	0/88

^a^ farms were all fattening; small < 2500 pigs housed; ^b^ prevalence; ^c^ Real-time-RT-PCR was performed on 88 plasma samples, i.e., 11 samples from each batch.

## Data Availability

Not applicable.
